# BézierCE: Low-Light Image Enhancement via Zero-Reference Bézier Curve Estimation

**DOI:** 10.3390/s23239593

**Published:** 2023-12-03

**Authors:** Xianjie Gao, Kai Zhao, Lei Han, Jinming Luo

**Affiliations:** 1 Department of Basic Sciences, Shanxi Agricultural University, Taigu 030801, China; xjgao@sxau.edu.cn; 2 Faculty of Engineering, University of New South Wales, Sydney, NSW 2052, Australia; kai.zhao2@student.unsw.edu.au; 3 School of Sciences, Harbin University of Science and Technology, Harbin 150080, China; leihan1998@126.com; 4 School of Mathematical Sciences, Dalian University of Technology, Dalian 116024, China

**Keywords:** low-light image enhancement, zero reference, Bézier curve

## Abstract

Due to problems such as the shooting light, viewing angle, and camera equipment, low-light images with low contrast, color distortion, high noise, and unclear details can be seen regularly in real scenes. These low-light images will not only affect our observation but will also greatly affect the performance of computer vision processing algorithms. Low-light image enhancement technology can help to improve the quality of images and make them more applicable to fields such as computer vision, machine learning, and artificial intelligence. In this paper, we propose a novel method to enhance images through Bézier curve estimation. We estimate the pixel-level Bézier curve by training a deep neural network (BCE-Net) to adjust the dynamic range of a given image. Based on the good properties of the Bézier curve, in that it is smooth, continuous, and differentiable everywhere, low-light image enhancement through Bézier curve mapping is effective. The advantages of BCE-Net’s brevity and zero-reference make it generalizable to other low-light conditions. Extensive experiments show that our method outperforms existing methods both qualitatively and quantitatively.

## 1. Introduction

With the rapid development of information technology and deep learning, image processing has become an indispensable and important technology in the application of the field of artificial intelligence, such as in medical images [1], image recognition [2], agricultural research [3], traffic information systems [4], object detection [5], and image segmentation [6]. During the image acquisition process, it is easy to produce a large number of low-light images under conditions such as low-light environments, low-end devices, and unreasonable camera equipment configurations. Low-light images will adversely affect people’s subjective visual experience and the performance of computer vision systems due to the shortcomings of color distortion, high noise, damaged quality, and low contrast. Therefore, the study of low-light image enhancement has strong practical significance.

Image enhancement can be used in all areas with low-light image scenarios, e.g., object detection [7], underwater images [8], underground utilities [9], autonomous driving [10], and video surveillance [11]. It is difficult or even impossible to achieve low-light image enhancement by changing the shooting environment or improving the hardware of the shooting equipment. Therefore, it is necessary to process images through low-light image enhancement algorithms.

In this paper, we propose a novel method to enhance images through Bézier curve estimation. The core idea of this paper is using Bézier representation to estimate the light enhancement curve. Instead of image-to-image mapping, our approach takes the low-light image as an input and estimates the parameters of the light enhancement curve; thus, we can dynamically adjust pixels to enhance the image. Using the good properties of the Bézier curve, such as its smoothness, continuousness, and differentiability, the image enhancement effect can be guaranteed. Similar to the existing methods [12,13,14], our method involves unsupervised learning and zero-reference during the training process; it does not require paired or even unpaired data, and it can be used in various dark-light environments, as well as having a good generalization performance. The experimental results demonstrate that our method outperforms existing methods in subjective feelings and objective indicators. The main contributions of this paper can be summarized as follows:

1. Based on the good properties of the Bézier curve, we use it as the output for the dynamic adjustment of pixels. Compared with Zero-DCE, we overcome the overexposure problem.
2. This paper proposes a zero-shot learning model with a short training time, which effectively avoids the risk of overfitting and improves the generalization ability.
3. Experiments on a number of low-light image datasets reveal that our method outperforms some of the current state-of-the-art methods.

The rest of the paper is organized as follows: In Section 2, we give an introduction to related works. In Section 3, we propose the zero-reference method to enhance images through Bézier curve estimation. Section 4 presents the experimental results, and the last section concludes the paper.

## 2. Related Works

In this section, we review the related works on low-light image enhancement, mainly including conventional methods (CMs) and deep learning methods.

### 2.1. Conventional Methods

Among conventional low-light image enhancement algorithms, the histogram equalization (HE) algorithms and algorithms based on the Retinex model are commonly used.

#### 2.1.1. Histogram Equalization Algorithms

The HE algorithm uses the image histogram to adjust the contrast of the image and improves the image contrast by uniformly expanding the concentrated gray range to the entire gray range [15,16,17]. However, during the HE processing, the contrast of the noise may be increased, and some useful signals may be lost. To overcome the inherent weaknesses of these shortcomings, many improved HE algorithms have been proposed [18,19,20,21,22,23].

#### 2.1.2. Retinex Model-Based Methods

Retinex theory is commonly used in image enhancement based on scientific experiments and analysis [24]. The method based on the Retinex model decomposes the low-light image *S* into reflection property *R* and illumination map *I*. After that, many kinds of improved versions of the Retinex model appeared, e.g., the single-scale Retinex model (SSR) [25], multiscale Retinex model (MSR) [26], variational Retinex model [27,28,29,30], and the maximum-entropy-based Retinex model [31]. However, the calculating velocity of the Retinex algorithm is relatively slow, and the computational complexity of the variational Retinex model is high. This method cannot be applied to some real-time low-light image enhancement scenes.

### 2.2. Deep Learning Methods

In recent years, low-light image enhancement based on deep learning methods has attracted widespread attention. Compared with conventional methods, deep-learning-based methods have better accuracy, better generalization ability, and faster computing speed. According to different learning strategies, low-light image enhancement methods based on deep learning can be divided into supervised learning (SL), unsupervised learning (UL), semi-supervised learning (SSL), reinforcement learning (RL), and zero-shot learning (ZSL). In the following subsections, we briefly review some representative approaches to these strategies.

#### 2.2.1. Supervised Learning

Lore et al. [32] proposed a pioneering work on low-light image enhancement based on deep learning. Subsequently, a variety of supervised-learning-based low-light image enhancement methods have been studied, e.g., the convolutional neural network [33], residual convolutional neural network [34], Msr-net [35], Retinex-Net [36], LightenNet [37], DeepUPE [38], KinD [39], EEMEFN [40], luminance-aware pyramid network [41], deep lightening network [42], and the progressive–recursive image enhancement network [43].

#### 2.2.2. Unsupervised Learning

To address the issue that training a deep model on paired data may lead to overfitting and limit the generalization ability, Jiang et al. [12] designed an unsupervised generative adversarial network without unpaired images. Fu et al. [44] proposed LE-GAN based on generative adversarial networks using an attention module and identity invariant loss. Xiong et al. [45] used decoupled networks for unsupervised low-light image enhancement. Han et al. [46] proposed unsupervised learning based on a dual-branch fusion low-light image enhancement algorithm.

#### 2.2.3. Semi-Supervised Learning

In order to combine the advantages of supervised learning and unsupervised learning, semi-supervised learning was proposed. Yang et al. [47] proposed a low-light image enhancement method using a deep recursive band network (DRBN). Chen et al. [48] put forward a semi-supervised network framework (SSNF) to enhance low-light images. Malik and Soundararajan [49] proposed semi-supervised learning for low-light image restoration via quality-assisted pseudo-labeling.

#### 2.2.4. Reinforcement Learning

Without paired training data, Yu et al. [50] proposed a method to enhance low-light images through reinforcement learning. Zhang et al. [51] presented a deep reinforcement learning method (ReLLIE) for customized low-light enhancement. Cotogni and Cusano [52] introduced a lightweight fully automatic and explainable method for low-light image enhancement.

#### 2.2.5. Zero-Shot Learning

In order to make up for the shortcomings of supervised learning, reinforcement learning, and unsupervised learning methods, the zero-shot learning method was proposed. Zhang et al. [53] proposed a zero-shot scheme for backlit image restoration that does not rely on any prior image examples or prior training. Zhu et al. [54] proposed a three-branch convolution neural network (RRDNet) for underexposed image restoration. Zhao et al. [55] combined Retinex-based and learning-based methods for low-light image enhancement. Liu et al. [56] proposed a Retinex-inspired Unrolling with cooperative prior architecture search for low-light image enhancement. Zheng and Gupta [57] introduced a novel semantic-guided zero-shot low-light image enhancement network implemented through enhancement factor extraction, recurrent image enhancement, and unsupervised semantic segmentation. Gao et al. [58] proposed a novel low-light image enhancement method via the Retinex composition of denoised Deep Image Prior. Xie et al. [59] proposed a zero-shot Retinex network (IRNet) composed of Decom-Net and Enhance-Net to alleviate the problem of low brightness and low contrast. In addition, depth curve estimation networks based on image reconstruction have been proposed. Guo et al. [14] presented an approach that treats light enhancement as an image-specific curve estimation task using deep networks. Li et al. [60] proposed Zero-DCE++, a fast and lightweight version of Zero-DCE. The novel low-light image enhancement method we propose in this paper also belongs to depth curve estimation networks.

## 3. Methodology

**Figure 1:**
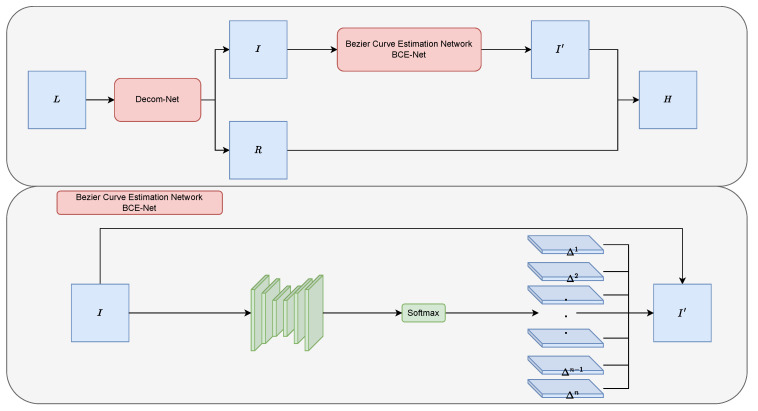
Overview of our method.

We present the framework of BézierCE in Figure 1. The Bézier Curve Estimation Network (BCE-Net) is devised to estimate the best fitting light enhancement curve given the input low-light image.

### 3.1. Decomposition

For a low-light image L, we first use a subnetwork Decom-Net to decompose the input image L into reflectance R and illumination I:

R,I=Decom−Net(L).

In practice, we make use of a CNN-architecture neural network to build the Decom-Net. According to the prior knowledge of computer vision, we use the shadow layers’ output as R and the deep layers’ output as I. We describe the network details in Table 1.

### 3.2. Bézier Curve Estimation

The inspiration comes from the curve adjustments used in photo editing. We try to design a parameter-controlled curve that can automatically map low-brightness images to their enhanced versions, where the adaptive curve parameters entirely depend on the input image. This curve design has three objectives: (1) Each pixel value of the enhanced image should be within the normalized range of [0,1] to avoid information loss due to overflow or truncation. (2) The curve should maintain unidirectionality to preserve the differences (contrast) between adjacent pixels. (3) The curve should be controlled by a class of parameter curves, and the control method should be as simple as possible.

In order to control the parametric curve, the simplest parametric curve in computational geometry is the Bézier curve. But, how do we maintain the unidirectionality to preserve the differences (contrast) between adjacent pixels? To solve this problem, we designed the curve to be controlled by *n* parameters $\Delta^{1},\Delta^{2},\Delta^{3},\cdots ,\Delta^{n}$; these *n* parameters should sum to 1: $\sum_{i}\Delta^{i}=1$. This implicitly defines the control points of the Bézier curve:

(1) $$P_{0}=\left(0,0\right),P_{1}=\left(\frac{1}{n},\Delta^{1}\right),\cdots ,P_{i}=\left(\frac{i}{n},\sum_{t=1}^{i}\Delta^{t}\right),\cdots ,P_{n}=\left(1,1\right).$$

Then, the Bézier curve controlled by these control points can be formulated as

(2) $$P_{t}=\sum_{i}^{n}C_{n}^{i}t^{i}{\left(1-t\right)}^{n-i}P_{i}.$$

Based on this formulation, we design the Bézier Curve Estimation Network to estimate the Bézier curve parameters of each pixel: $\Delta_{x,y}^{t}$. The $\Delta_{x,y}^{t}$ represents the parameter of the *t*-th control point for the pixel at position (x,y) on the image. The output illumination $I^{\prime }$ can be formulated as

(3) $$I^{\prime }\left(x,y\right)=\sum_{i}^{n}C_{n}^{i}\sum_{t=1}^{i}\Delta_{x,y}^{i}{\left(I\left(x,y\right)\right)}^{i}{\left(1-I\left(x,y\right)\right)}^{n-i},$$

where $I{\left(x,y\right)}^{i}$ represents the *i*-th power of the image intensity I at a specific pixel coordinate (x,y). The output enhanced image can be expressed as

(4) $$H=I^{\prime }\times R.$$

Table 2 describes the network details.

### 3.3. Non-Reference Loss Functions

To enable zero-reference learning in BézierCE, we used Spatial Consistency Loss, Exposure Control Loss, Color Constancy Loss, and Illumination Smoothness Loss in our experiments. We briefly introduce these loss functions.

#### 3.3.1. Spatial Consistency Loss

By preserving the differences between adjacent regions in the input image and its enhanced version, the spatial consistency loss ($L_{spa}$) is defined as

(5) $$L_{spa}=\frac{1}{K}\sum_{i=1}^{K}\sum_{j\in \Omega (i)}{\left(\left|\left(H_{i}-H_{j}\right)\right|-\left|\left(L_{i}-L_{j}\right)\right|\right)}^{2},$$

where *K* and Ω(i), respectively, denote the number of local regions and the four neighboring regions (top, down, left, and right) centered on region *i*. H represents the average intensity values of the local region in the enhanced version, and L represents the input image. The size of the local region is empirically set to 4×4. It is worth noting that the loss remains stable regardless of other region sizes.

#### 3.3.2. Exposure Control Loss

In order to restrain underexposed/overexposed areas, the exposure control loss ($L_{exp}$) is designed to control the exposure level. In the experiment, *E* is set to 0.6, and the loss $L_{exp}$ can be expressed as

(6) $$L_{\exp}=\frac{1}{M}\sum_{k=1}^{M}\left|H_{k}-E\right|,$$

where *M* denotes the count of nonoverlapping local regions with a size of 16×16. *H* has the same meaning as in the spatial consistency loss.

#### 3.3.3. Color Constancy Loss

In order to correct the potential color deviations in the enhanced image and establish the relationship between the three adjustment channels, the color constancy loss ($L_{col}$) can be mathematically represented as

(7) $$L_{col}=\sum_{\forall (m,n)\in \varepsilon }{\left(J^{m}-J^{n}\right)}^{2},\varepsilon =\left\{\left(R,G\right),\left(R,B\right),\left(G,B\right)\right\},$$

where $J_{m}$ represents the average intensity value of the *m*-th channel in the enhanced image, and (m,n) denotes a pair of channels.

#### 3.3.4. Illumination Smoothness Loss

To maintain the smoothness and monotonicity relationships between adjacent pixels, an illumination smoothness loss ($L_{tv}$) is added for each curve parameter map A, which is defined as

(8) $$L_{tv}=\frac{1}{N}\sum_{n=1}^{N}\sum_{c\in \xi }{\left(\left|\nabla_{x}A_{n}^{c}\right|+\nabla_{y}A_{n}^{c}|\right)}^{2},\xi =\left\{R,G,B\right\},$$

where *N* represents iterations, and ∇x and ∇y denote the horizontal and vertical gradient operations, respectively.

#### 3.3.5. Total Loss

The total loss is the combination of these four loss functions:

(9) $$L=\lambda_{spa}L_{spa}+\lambda_{exp}L_{exp}+\lambda_{col}L_{col}+\lambda_{tv}L_{tv},$$

where $\lambda_{spa}$, $\lambda_{exp}$, $\lambda_{col}$, and $\lambda_{tv}$ are the weights of the four losses.

## 4. Experiment

In this section, we present the performance results of our approach and five representative methods on five public low-light image datasets and also introduce the experimental setup and performance metrics.

### 4.1. Training Setting

To take full advantage of the capability of wide dynamic range adjustment, low-light and over-exposed images were included in the training set. The training method was consistent with reference [14]. DCE-Net was trained using 360 multi-exposure sequences from Part1 of the SICE dataset [61]. These 3022 images of different exposure levels in the Part1 subset [61] were randomly divided into two parts, including 2422 images used for training and the rest used for validation. Prior to training, the images were resized to 512×512 pixels.

We implemented our framework with PyTorch on an NVIDIA 2080Ti GPU. The batch size, and weights $\lambda_{spa}$, $\lambda_{exp}$, $\lambda_{col}$, and $\lambda_{tv}$ were set to 8, 1, 10, 5, and 200, respectively. Bias was initialized to a constant value. The filter weights of each layer were initialized using a Gaussian function with standard zero mean and 0.02 standard deviation. Optimization was performed using the Adam optimizer with default parameters and a fixed learning rate η=0.0001.

### 4.2. Performance Criteria

In addition to measuring the experimental results by visual observation, we also used the following evaluation indicators of the no-reference evaluation method.

**Natural image quality evaluator (NIQE).** The NIQE evaluation index is biased towards the evaluation of image naturalness, clarity, and noise [62]. A lower NIQE score indicates that the naturalness of the enhanced image is better preserved.

**Colorfulness-based Patch-based Contrast Quality Index (CPCQI).** CPCQI is a color-based contrast quality evaluation index [63]. A larger CPCQI value indicates a higher enhanced image effect.

### 4.3. Results

In this subsection, in addition to showing our method for low-light image enhancement, there are five other representative methods, respectively, LIME [64], NPE [65], SRIE [66], KinD [39], and Zero-DCE [14] (three conventional methods and two deep learning methods). The enhancement effects of these methods are compared qualitatively and quantitatively on five low-light image datasets, namely DICM [21], LIME [64], MEF [67], NPE [65], and VV (https://sites.google.com/site/vonikakis/datasets, accessed on 5 September 2023).

#### 4.3.1. Qualitative Evaluation

**Figure 2:**
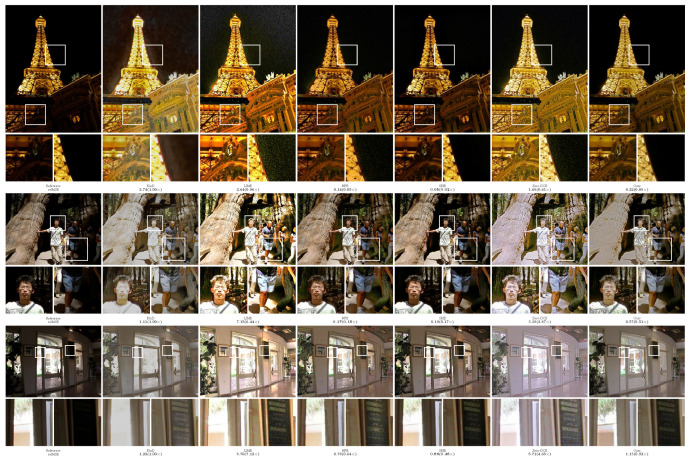
Comparisons of enhanced images on the DICM dataset.

**Figure 3:**
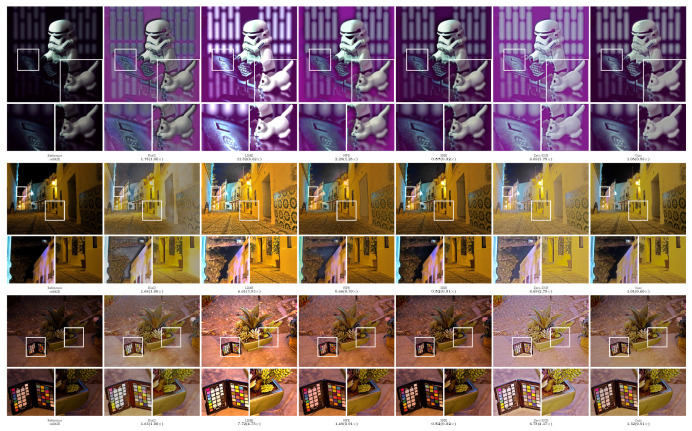
Comparisons of enhanced images on the LIME dataset.

**Figure 4:**
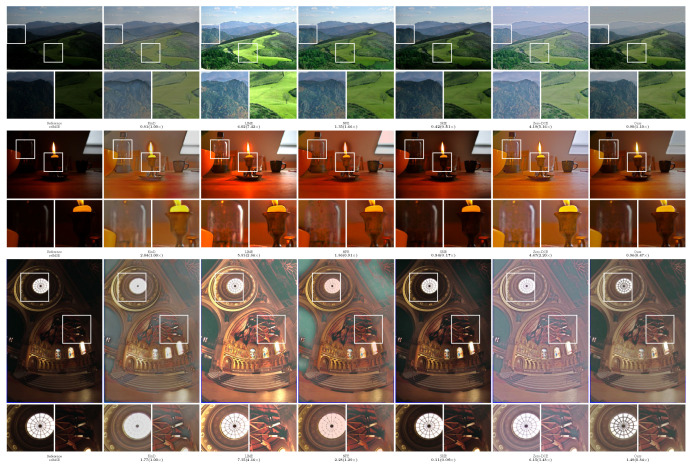
Comparisons of enhanced images on the MEF dataset.

**Figure 5:**
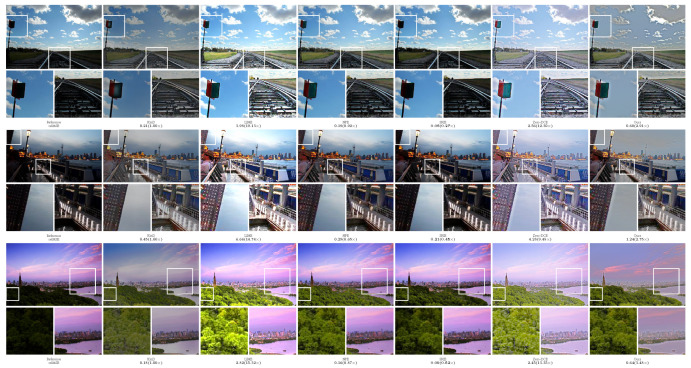
Comparisons of enhanced images on the NPE dataset.

**Figure 6:**
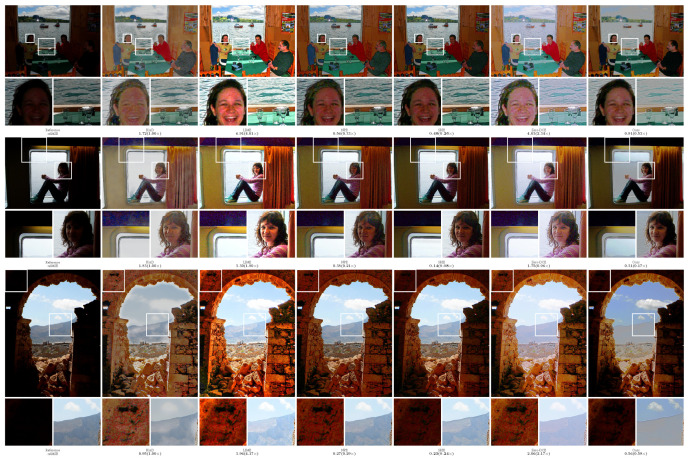
Comparisons of enhanced images on the VV dataset.

In order to more intuitively compare the performance of different methods, the experiment provided the visualization results of the enhanced images, as shown in Figure 2, Figure 3, Figure 4, Figure 5 and Figure 6. The boxes in the figure represent local area enlargements to better demonstrate the obvious differences. As can be seen in Figure 2, the brightness enhancement effect of the NPE and SRIE methods is not obvious. The local over-brightness of the KinD, LIME, and Zero-DCE methods leads to a decrease in the naturalness of the image and color distortion. As shown in Figure 3, the LIME and Zero-DCE methods are overexposed. KinD has color distorted, and the NPE and SRIE enhancement effects are not obvious. As can be seen from the circular structure of the roof in Figure 4, the image becomes blurred after enhancement by the KinD and NPE methods. The enhancement effect of the SRIE method is not obvious, and the color is distorted by the LIME and Zero-DCE. Corresponding conclusions can also be obtained from the observations in Figure 5 and Figure 6. From the observation in Figure 2, Figure 3, Figure 4, Figure 5 and Figure 6, our method can effectively enhance low-light images. The low-light images enhanced by our method have better naturalness and contrast, without the problems of overexposure and artifacts.

#### 4.3.2. Quantitative Comparison

In order to verify the performance of the algorithm in this paper, LIME, NPE, SRIE, KinD, and Zero-DCE were used as the comparison methods, respectively. LIME, NPE, and SRIE are conventional methods, KinD is a supervised learning method, and Zero-DCE is zero-shot learning method. The NIQE metrics of different methods on the five datasets are shown in Table 3. The CPCQI indicators of different methods on the five datasets are shown in Table 4. The red and magenta scores represent the top two in the corresponding dataset, respectively. Our method achieved the best NIQE results on the MEF and NPE datasets and the second-best result on LIME. Except for the DICM dataset, the NIQE results of our method were better than the Zero-DCE. Moreover, the NIQE results of our method were better than the KinD on all datasets. Our method achieved the second-best CPCQI results on the LIME and MEF. The CPCQI results of our method outperformed the two deep learning methods (KinD and Zero-DCE) on all datasets. It is worth noting that as a zero-shot learning method, our method was almost always better than Zero-DCE. This shows that the images enhanced by our method maintain better naturalness and contrast.

As shown in Table 5 and Table 6, we also compared the variance in the NIQE and CPCQI for different methods. Our method also performed well in the variance comparisons.

We also calculated the relMSE for low-light images and enhanced images, as shown in Figure 2, Figure 3, Figure 4, Figure 5 and Figure 6. RelMSE is the abbreviation of the relative mean square error, which is used to measure the reconstruction quality of the original image by the enhancement algorithm. This metric can reflect that our method has a better image reconstruction quality than Zero-DCE. Our method is slightly larger than SRIE and smaller than other methods in most cases. Combined with the visual effects, it can be seen that we do not enhance the originally dark areas too much, and the bright areas are not overexposed.

### 4.4. Ablation Study

**Figure 7:**
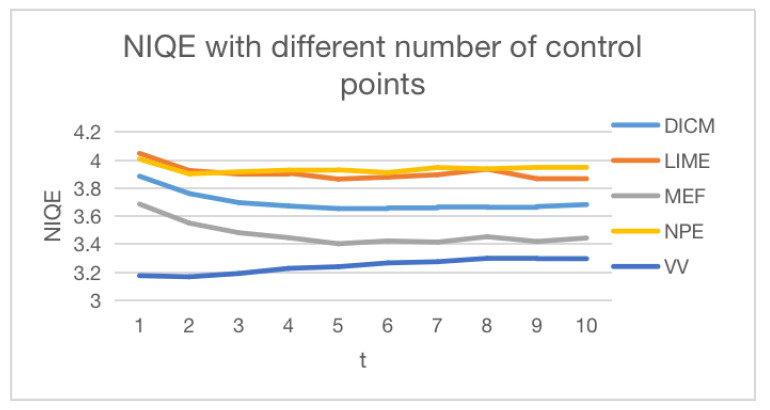
NIQE comparisons of different numbers of control points.

We analyzed our approach by comparing different models based on varying numbers of control points, as shown in Figure 7. We observe that using approximately five control points yielded good performance on different datasets. As the number of control points increases, the NIQE decreases for different datasets. This is consistent with our understanding that more control points should lead to better model performance. We finally chose five control points for the experiment. The reason the NIQE slightly increased on the VV dataset is because many images in the VV dataset were overexposed.

### 4.5. Time Analysis

As shown in Table 7, we compared the running time of different methods with five different input image sizes. Our approach is the most efficient method compared with other methods on different input image sizes. Unlike conventional methods such as LIME, NPE, and SRIE, the running time of our method changes very little as the image resolution increases. Compared with the KinD, the memory of our method does not increase significantly with the increase in the image resolution.

**Figure 8:**
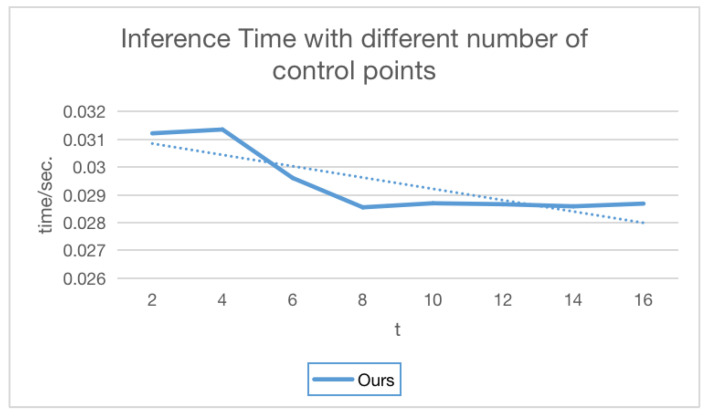
Inference time comparisons of different numbers of control points.

We also compared the inference time of our method based on different numbers of control points. As shown in Figure 8, as the number of control points increases, the inference time of our method does not increase significantly. In Figure 8, the blue solid line is the inference time, and the blue dotted line is the trend.

## 5. Conclusions

In this paper, we proposed a novel method called BézierCE, which builds upon the Zero-DCE algorithm by introducing control points to manipulate the curve at different locations. To determine the parameters of these control points, we employed a neural network with a U-net architecture to approximate their positions, allowing us to generate a Bézier curve based on these control points. In our experiments, we observed significant improvements in mitigating overexposure issues compared to Zero-DCE. In addition, unlike the iterative adjustments in Zero-DCE, our method offers faster processing speeds during testing because we adjust the parameters of the curve in one regression step.

However, our approach faces certain limitations. Compared to Retinex-based methods, our approach accurately determines the gamma value for individual pixels based on the prior information of the entire image. We acknowledge that our method currently struggles with noise situations. We plan to address this limitation in our future work, aiming to enhance its performance. The enhancement of brightness may not be as pronounced, especially in situations where the global illumination is very dim. Addressing this issue may require incorporating constraints based on our experience with dark images. Furthermore, we are also considering exploring block-wise adjustments for images, which will be the focus of our future work.

## Figures and Tables

**Table 1:** Decom-Net architecture.

Layer	Params	Input Dim	Output Dim	Activate Function	Input Layer
Max	-	(3,H,W)	(1,H,W)	-	Input
Conv0	(4,64,9,9)	(4,H,W)	(64,H,W)	ReLU	Cat (Input,Max)
Conv1	(64,64,3,3)	(64,H,W)	(64,H,W)	ReLU	Conv0
Conv2	(64,64,3,3)	(64,H,W)	(64,H,W)	ReLU	Conv1
Conv3	(64,64,3,3)	(64,H,W)	(64,H,W)	ReLU	Conv2
Conv4	(64,64,3,3)	(64,H,W)	(64,H,W)	ReLU	Conv3
Conv5	(64,64,3,3)	(64,H,W)	(64,H,W)	ReLU	Conv4
Conv6	(64,4,3,3)	(64,H,W)	(4,H,W)	Sigmoid	Conv5
Split	-	(4,H,W)	R:(3,H,W); I:(1,H,W)	-	Conv6

**Table 2:** BCE-Net architecture.

Layer	Params	Input Dim	Output Dim	Activate Function	Input Layer
Conv0	(1,32,3,3)	(1,H,W)	(32,H,W)	ReLU	Input
Conv1	(32,32,3,3)	(32,H,W)	(32,H,W)	ReLU	Conv0
Conv2	(32,32,3,3)	(32,H,W)	(32,H,W)	ReLU	Conv1
Conv3	(32,32,3,3)	(32,H,W)	(32,H,W)	ReLU	Conv2
Conv4	(64,32,3,3)	(64,H,W)	(32,H,W)	ReLU	Cat(Conv2,Conv3)
Conv5	(64,32,3,3)	(64,H,W)	(32,H,W)	ReLU	Cat(Conv1,Conv4)
Conv6	(64,t,3,3)	(64,H,W)	(t,H,W)	-	Cat(Conv0,Conv5)
Softmax	-	-	-	-	Conv6

**Table 3:** Comparison of the average NIQE on five datasets.

Learning	Method	DICM	LIME	MEF	NPE	VV
CM	LIME	3.5360	4.1423	3.7022	4.2625	2.7475
	NPE	3.4530	3.9031	3.5155	3.9501	3.0290
	SRIE	3.5768	3.7868	3.4742	3.9883	3.1357
SL	KinD	4.2691	4.3525	4.1318	3.9589	3.4255
ZSL	Zero-DCE	3.6091	3.9354	3.4044	4.0944	3.2245
	Ours	3.6334	3.8553	3.3939	3.9021	3.1680

**Table 4:** Comparison of the average CPCQI on five datasets.

Learning	Method	DICM	LIME	MEF	NPE	VV
CM	LIME	0.8986	1.0882	1.0385	0.9844	0.9555
	NPE	0.9139	1.0812	1.0372	1.0228	0.9557
	SRIE	0.9056	1.1121	1.0967	1.0258	0.9629
SL	KinD	0.7459	0.8336	0.7877	0.8007	0.7418
ZSL	Zero-DCE	0.7818	0.9803	0.9461	0.8578	0.8396
	Ours	0.8591	1.1016	1.0544	1.0135	0.9402

**Table 5:** Comparison of the variance NIQE on five datasets.

Learning	Method	DICM	LIME	MEF	NPE	VV
CM	LIME	1.6156	5.7242	0.8649	1.5840	0.4898
	NPE	1.8238	3.8316	1.2291	1.5272	0.5843
	SRIE	1.7111	2.6850	0.8935	1.0207	0.7014
SL	KinD	1.2968	2.9960	0.5795	1.9315	0.5780
ZSL	Zero-DCE	2.1590	5.1292	1.0877	0.9627	0.5733
	Ours	2.2209	2.5713	1.0417	1.1559	0.7503

**Table 6:** Comparison of the variance CPCQI on five datasets.

Learning	Method	DICM	LIME	MEF	NPE	VV
CM	LIME	0.0117	0.0138	0.0097	0.0040	0.0043
	NPE	0.0064	0.0210	0.0154	0.0062	0.0045
	SRIE	0.0034	0.0123	0.0082	0.0112	0.0052
SL	KinD	0.0061	0.0153	0.0043	0.0093	0.0032
ZSL	Zero-DCE	0.0150	0.0209	0.0135	0.0108	0.0102
	Ours	0.0022	0.0091	0.0035	0.0043	0.0032

**Table 7:** Runtime (RT) comparisons (in seconds).

Method	(640×480)	(1280×960)	(1920×1440)	(2560×1920)	(3200×2400)
LIME	0.1133	0.4196	1.0148	1.5713	2.3901
NPE	5.8861	26.6340	58.5019	104.8345	163.9938
SRIE	4.7643	33.6684	121.5802	343.9839	726.5981
KinD	0.1554	0.0464	-	-	-
Zero-DCE	0.12559	0.1390	0.2539	0.4051	0.83371
Ours	0.0301	0.0325	0.0724	0.1192	0.1882

## Data Availability

Data are contained within the article.
